# Optimization of In Vivo Studies by Combining Planar Dynamic and Tomographic Imaging: Workflow Evaluation on a Superparamagnetic Nanoparticles System

**DOI:** 10.1155/2021/6677847

**Published:** 2021-01-15

**Authors:** Maritina Rouchota, Alessio Adamiano, Michele Iafisco, Eirini Fragogeorgi, Irineos Pilatis, Gilles Doumont, Sébastien Boutry, Daniele Catalucci, Argyro Zacharioudaki, George C. Kagadis

**Affiliations:** ^1^3dmi Research Group, Department of Medical Physics, School of Medicine, University of Patras, Greece; ^2^Institute of Science and Technology for Ceramics (ISTEC), National Research Council (CNR), Italy; ^3^Institute of Nuclear & Radiological Sciences, Technology, Energy & Safety, NCSR “Demokritos”, Greece; ^4^Department of Biomedical Engineering, University of West Attica, Greece; ^5^Center for Microscopy and Molecular Imaging (CMMI), Université Libre de Bruxelles (ULB), Rue Adrienne Bolland 8, B-6041 Charleroi (Gosselies), Belgium; ^6^Institute of Genetic and Biomedical Research (IRGB), National Research Council (CNR), UOS Milan, Italy; ^7^Humanitas Clinical and Research Center, IRCCS, Rozzano (Milan), Italy; ^8^DVM MLAS Dipl.ECLAM, Greece

## Abstract

Molecular imaging holds great promise in the noninvasive monitoring of several diseases with nanoparticles (NPs) being considered an efficient imaging tool for cancer, central nervous system, and heart- or bone-related diseases and for disorders of the mononuclear phagocytic system (MPS). In the present study, we used an iron-based nanoformulation, already established as an MRI/SPECT probe, as well as to load different biomolecules, to investigate its potential for nuclear planar and tomographic imaging of several target tissues following its distribution via different administration routes. Iron-doped hydroxyapatite NPs (FeHA) were radiolabeled with the single photon *γ*-emitting imaging agent [^99m^Tc]TcMDP. Administration of the radioactive NPs was performed via the following four delivery methods: (1) standard intravenous (iv) tail vein, (2) iv retro-orbital injection, (3) intratracheal (it) instillation, and (4) intrarectal installation (pr). Real-time, live, fast dynamic screening studies were performed on a dedicated bench top, mouse-sized, planar SPECT system from *t* = 0 to 1 hour postinjection (p.i.), and consequently, tomographic SPECT/CT imaging was performed, for up to 24 hours p.i. The administration routes that have been studied provide a wide range of possible target tissues, for various diseases. Studies can be optimized following this workflow, as it is possible to quickly assess more parameters in a small number of animals (injection route, dosage, and fasting conditions). Thus, such an imaging protocol combines the strengths of both dynamic planar and tomographic imaging, and by using iron-based NPs of high biocompatibility along with the appropriate administration route, a potential diagnostic or therapeutic effect could be attained.

## 1. Introduction

Contemporary drug development in the era of personalized medicine has made molecular targeting and genomics key players, in the place of previously empiric screening of biologically active compounds [1]. Towards this approach, the most prominent new compounds for targeting are evolving to nanoparticles (NPs), peptides, and antibodies with specific characteristics [2–5].

*In vivo* testing constitutes a critical part of preclinical development of these compounds, as it provides the first evidence of any favorable effect on a whole organism, thus taking into account complex mechanisms. In many cases, the exact biodistribution of these compounds, as well as their targeting efficacy, is severely affected by the administration route of choice [6]. In particular, the choice of the administration route is closely correlated to the type of disorder studied, for diagnostic and/or therapeutic purposes. The most common administration routes are subcutaneous, intraperitoneal, or intravenous injection (both tail vein and retro-orbital) [7]. The absorption rate differs for each route, in general changing from highest to lowest in the following way: intravenous (iv), intraperitoneal (ip), intramuscular (im), subcutaneous (sc), and orally (po) [8]. Thus, choosing the most appropriate administration route for a given application is of great importance.

Over the last decade, NPs were extensively studied as carriers of drugs to improve pharmacodynamics and to reduce their side effects. Moreover, a wide number of NPs have been proposed as diagnostic agents for Magnetic Resonance Imaging (MRI), such as superparamagnetic iron oxide NPs (SPIONs) and doped calcium phosphates [9]. In this respect, it has been proven that different routes of NPs' administration could lead to varying effects on the tissue distribution, biodegradation, metabolism, and elimination [10] [11]. More specifically, following local administration, NPs remain close to the site of injection and are eventually excreted through the lymphatic system, if their size is in the range between 10 and 60 nm as lymphatic uptake is size-dependent. NPs' size also plays a critical role when subcutaneously injected, as NPs with a diameter less than 120 nm could pass through the lymph nodes to the bloodstream [11]. When given orally, they are concentrated on the gastrointestinal tract and are eliminated via feces. Intravenously injected NPs with a hydrodynamic diameter larger than 8 nm [12] have a tendency to localize at the vascular system and in particular at the organs of the mononuclear phagocyte system (MPS) (i.e., liver, spleen, and kidneys) [13], while smaller NPs are cleared via the urinary bladder [14]. These points also highlight the effect that different sizes but also hydrophilicity or lipophilicity and targeting moieties have in the final biodistribution of the compounds in vivo. Targeting moieties can change the biodistribution of NPs, and size differences can alter the biodistribution and eventually the kinetic of the NPs. In fact, the uptake by macrophage of larger NPs is quicker with respect to smaller ones that will circulate in the blood flow for longer times. This also means that the accumulation of NPs in the organs of the mononuclear phagocyte system is faster for larger particles. These effects have to be taken into account when performing in vivo experiment using different injection routes with NPs with different sizes or functionalized with different targeting molecules.

Molecular imaging holds great promise in the noninvasive monitoring of several diseases with NPs being considered as an efficient imaging tool for cancer, central nervous system, heart- or bone-related diseases, and for disorders of the MPS [13, 15, 16]. Regarding diagnostic applications, when using NPs for the treatment or the monitoring of some physiological or pathological conditions like chronic kidney failure [17], it is preferable that they are rather quickly metabolised and eliminated, to avoid any toxic effects [13]. SPIONs are one of the most prominent multimodal probes in biomedical imaging, as they are considered a versatile diagnostic tool for various pathologies, such as cancer, diseases of lymphatic system, central nervous system, cardiovascular system, and infectious diseases [15, 18].

One of the most common approaches to enable the study of the biodistribution of these NPs is by labeling them with fluorescent tag or radionuclide to enable optical and nuclear imaging. Optical probe or radioactive probe itself means the whole tracer including the tag as such and does not refer just to the tag molecule [19, 20]. Labeling with fluorescent tag and monitoring with optical imaging are very popular and target specific, but it is not quantitative, due to signal attenuation and scatter by adjacent tissues and cannot be used in deep located structures, just on superficial tumours or structures or in surgeries [21]. On the other hand, nuclear labeling and imaging has the advantage of good tissue penetration and very low scatter from adjacent tissues, providing both quantitative results and the ability for tomographic imaging [21]. Methods of radiolabeling NPs able to avoid any alteration in the chemical and physical properties that may impact on their pharmacologic profile of the NPs have been extensively studied [22, 23].

Biomolecules such as peptides or antibodies have been well studied over the past decades, and existing literature can be used as a reference to provide a roadmap on the *in vivo* evaluation of any new compound. On the contrary, there are no established protocols or workflows for systematic testing of new NPs, especially when targeted for new applications. Most of the literature studies show that the preliminary evaluation of their therapeutic action is conducted mainly by *in vitro* and *ex vivo*, while imaging is only introduced at the final stages of their evaluation.

In this study, we propose and evaluate an imaging workflow that combines the strengths of both planar dynamic and tomographic radioisotopic imaging as an efficient approach to optimize preclinical NPs testing. A magnetic iron doped hydroxyapatite (FeHA) NP formulation has been assessed, as a promising imaging tool for different target sites, depending on the administration route of choice. This formulation was initially developed for use as an MRI contrast agent and was then enriched to incorporate additional possibilities to enable multimodal and complementary imaging [24]. Alternative administration routes have been tested through 2D scintigraphic and 3D SPECT imaging on mice, to evaluate their ability to effectively target different tissues.

## 2. Materials and Methods

### 2.1. FeHA Synthesis

The synthesis and the physicochemical properties of superparamagnetic iron doped hydroxyapatite (FeHA) NPs have been extensively described in previous works (Iannotti 2017.). Briefly, for the synthesis, 10.64 g of H_3_PO_4_ (>85 wt% in water, Sigma-Aldrich, St. Louis, MO, USA) was dissolved in 35 mL of ultrapure water and added dropwise into a Ca(OH)_2_ (ACS reagent ≥ 99.0%, Sigma-Aldrich, St. Louis, MO, USA) suspension (12.0 g in 60 mL) containing FeCl_2_·4H_2_O (ACS reagent ≥ 99.0%, Sigma-Aldrich, St. Louis, MO, USA) (3.08 g) and FeCl_3_·6H_2_O (ACS reagent ≥ 99.0%, Sigma-Aldrich, St. Louis, MO, USA) (4.24 g) in a 1 : 1 molar ratio at 45°C under vigorous stirring. After the addition of phosphoric acid was completed, the obtained solution was kept at 45°C under stirring for 3 h and then left still at room temperature overnight. FeHA in the form of powder was recovered by centrifugation (6000 rpm, 5 min, 4°C) of the reaction mixture, repeatedly rinsed with water and finally freeze-dried before any further step.

### 2.2. Radiolabeling of FeHa

FeHA NPs were radiolabeled with the single photon *γ*-emitting metastable isotope of technetium, Tc (^99m^Tc), following the radiolabeling methodology as applied in our previous work [24]. In brief, the reaction took place at a high concentration of NPs to avoid centrifugation for the removal of free radioisotope, which could cause formation of aggregates. Therefore, an aliquot of 25-50 *μ*L of [^99m^Tc]Tc MDP (~15-19 MBq) was added to 500 *μ*L ^Fe^CaPs suspension (10 mg mL^−1^), and the mixture was allowed to react at room temperature under constant stirring for 30 min. Quality control of [^99m^Tc]*Τ*cMDP- FeHA was done with ITLC-Silica Gel (SG) (Agilent, US) using saline buffer (NaCl 0.9 wt%) as the mobile phase. ITLC analysis was performed on a Scan-RAM radio TLC detector (LabLogic Systems Ltd., (UK)). The chemicals and reagents used were of analytical grade. To ensure the serum stability of NPs in vivo, in vitro stability assays were first performed, at 0 min, 1 h, 3 h, and 24 h postconjugation, over a range of temperatures (at 5°C, 25°C, and 37°C) and in different media, namely, isotonic saline solution and human and bovine fetal serums at 37°C. The time-dependent increase of any free radioligand of ^99m^Tc-MDP was determined by using saline as the mobile phase system in Whatman 3MM or ITLC-SG strip.

All manipulations with gamma emitting radionuclides and their solutions were performed in areas with sufficient shielding by trained personnel in facilities supervised by the Greek Atomic Energy Commission and in compliance to national and international radiation-safety guidelines.

### 2.3. Magnetic Characterisation and Proof of Multimodal Functionality for FeHA NPs

A screening of FeHA contrast abilities in aqueous solution was conducted at five different iron concentrations from 0.002 mM to 0.15 mM to identify the optimal conditions for the in vivo experiments, similarly to what is already reported in the literature about liver MRI imaging with SPIONs [25, 26]. This procedure has been described in previously published work by the authors [24].

A preliminary in vitro experiment was conducted on a 7 Tesla MRI scanner (Bruker, BioSpec 70/30 USR, Paravision 5.1), equipped with 450/675 mT m^−1^ gradients (slew-rate: 3400-4500 T/m/s; rise-time 140 *μ*s) and a circular polarized mouse body volume coil with an inner diameter of 40 mm, using a multislice multiecho (MSME) sequence with the following parameters: repetition time (TR) = 2500 ms, 16 echos registered separately with first echo time (TE1) = 10.73 ms and echo spacing = 10.73 ms, field of view (FOV) = 20 mm × 40 mm, spatial resolution = 0.078 × 0.208 mm^2^/pixel, and scan time = 8 min. MR images were collected using MSME, fast low-angle shot gradient echo sequence (2D-FLASH), and Rapid Acquisition with Relaxation Enhancement (RARE) T2-weighted sequences.

For the in vivo imaging part, an MRI (Bruker 9.4 T) system was used, and images were acquired at 10 min and 60 min p.i. Images were collected using MSME, fast low-angle shot gradient echo sequence (2D-FLASH), and Rapid Acquisition with Relaxation Enhancement (RARE) T2-weighted sequences, with the following parameters: repetition time (TR) = 2,500 ms, 12 echos registered separately with first echo time (TE1) = 8.75 ms and echo spacing = 8.75 ms, spatial resolution = 0.177 × 0.180 mm^2^/pixel, and scan time = 8 min 20 s, 18 slices. For the 2D-FLASH is as follows:TR = 450 ms,TE = 3.2 ms, flip angle = 30°, spatial resolution = 0.133 × 0.094 mm^2^/pixel, and scan time = 2 min52 s 800 ms, 24 slices. Lastly, for the RARE T2 is as follows:TR = 3,500 ms,TE = 25.5 ms, spatial resolution = 0.133 × 0.08 mm pixel − 1, andscan time = 3 min15 s, 24 slices. The difference in contrast induction is presented by comparing the T2 relaxation times, pre- and postadministration of the FeHA NPs.

The exact same animals were imaged right after the MRI scans, on a SPECT/CT system (NanoSPECT/CT by Mediso, Hungary), further highlighting the direct multimodal applicability of the FeHa NP imaging. The acquisition duration for these two measurements lasted 1 hr and 1.5 hrs, respectively, and the images were reconstructed with 250 *μ*m voxel size.

### 2.4. Animals and Dosages

For the MRI studies, to further establish the contrast induction of the FeHA, two C57BL/6 healthy male mice (4 weeks old; 22-25 g) were used. Imaging was performed at 10 min and 60 min p.i. (tail vein injection) following the protocol described above. Animals were sacrificed at these time points, to stop any FeHA NPs kinetics that would affect MR imaging.

For the SPECT/CT and molecular screening studies, female Swiss-Webster Albino mice (4-6 weeks old; 20-30 g) were obtained from the breeding facilities of the National Center for Scientific Research “Demokritos” in Athens, Greece, and housed in an environment with controlled temperature (22°C), humidity, and a 12 h light/dark cycle, in individually ventilated cages. The mice were fed standard chow and tap water ad libitum and allowed to acclimate for 1 week. The protocol and all the animal procedures were approved by the General Directorate of Veterinary Services (Athens, Attica Prefecture, Greece) and by the Bioethical Committee of the Institution (Permit number: EL 25 BIO 022) on the basis of the European Directive 2010/63/EU on the protection of animals used for experimental purposes.

Anesthetization was performed with isoflurane anesthesia for all procedures. Levels of isoflurane ranged between 3 and 5% for anesthesia induction and 1-3% for anesthesia maintenance, during the administrations and the imaging acquisitions. For the intratracheal administration only, anesthetization was performed intraperitoneally (i.p.) with 100 *μ*L/10 g body weight of a stock solution containing 10% ketamine–hydrochloride (100 mg/mL) and 5% xylazine–hydrochloride (20 mg/mL), as suggested by relevant protocols [27]. Animal heating was ensured during all procedures that required the mice to remain anesthetised.

Administration of the NP solution was performed via one of the four following delivery methods: (1) standard IV tail vein, (2) intratracheal instillation, (3) IV retro-orbital injection, and (4) administration per rectum (PR). The administered solution had an initial activity of 1 mCi mL^−1^ and a NP concentration of 10 mg/mL. The administered volumes ranged between 50 and 150 *μ*L, depending on the route, which resulted in administered substance on the range of 0.5–1 mg of magnetic NPs per mouse. Briefly, for the standard IV injection, the lateral tail vein was cannulated with a 27-gauge needle, and a bolus injection of 150 *μ*L was given. For the intratracheal administration, a direct deposition to the lungs was performed by placing the animal on an angled platform, hanging by its incisors and by using a 22-gavage needle. A volume of 50 *μ*L was administered, followed by a 100 *μ*L air pocket behind the inoculum to ensure that all of the fluid is instilled into the lung [27]. For the retro-orbital IV injection of the venous sinus, the mouse was first anesthetized under 3-5% isoflurane and while still being unconscious, was injected with 150 *μ*L through a 30-gauge insulin needle [28]. PR administration was accomplished by using a PE tubing (0.28 mm ID × 0.61 mm OD × 25 mm) of 3^″^ length and a 30-gauge needle attached to its end. A bolus injection of 50 *μ*L was given [29].

All animal experiments were carried out in compliance with European and national regulations and after approval of protocols by national authorities.

### 2.5. In Vivo Molecular Screening

Real-time, live, fast dynamic screening studies were performed right after injection, on a dedicated bench top, mouse-sized, planar scintigraphic system (*γ*-eye™ by BIOEMTECH, Athens, Greece) [30]. The system also supports fusion with a digital mouse photograph, for anatomical coregistration. The main detector is based on two position-sensitive photomultiplier tubes, coupled to a CsI(Na) pixelated scintillator and a medium-energy lead collimator with parallel hexagonal holes that supports a range of SPECT isotopes. The system's field of view is 5 × 10 cm^2^, with spatial resolution of ~2 mm.

For the planar imaging, mice were kept under isoflurane anesthesia and under constant temperature of 37°C. A total of five animals (*n* = 5) were used for each administration route. Acquisitions started right after injection and had a total duration of 1 hour, separated in 2 min time frames, which allows real-time, live imaging of the substance kinetics. These dynamic acquisitions are exported in a video format, showing the biodistribution of the substance for the chosen time window (i.e., 1 hour). Additional short static scans are possible at different time points, i.e., 4 h or 24 h, to provide longitudinal information on the NP distribution on the same animal, requiring short anesthesia times, i.e., for 10 min or less.

### 2.6. SPECT/CT Imaging

Tomographic SPECT/CT imaging was performed with y-CUBE™ and x-CUBE™ (Molecubes, Belgium), respectively, after the first hour p.i. and then at 4 h and 24 h p.i. The SPECT system provides a spatial resolution of 0.6 mm for mouse imaging and of 1.5 mm for rat imaging. The CT system performs a spiral scan; it can provide images with 50 *μ*m resolution, and it operates between 35 and 80 kVp, 10-500 *μ*A tube current.

Mouse imaging was performed by keeping the mice anesthetized under isoflurane and under constant temperature of 37°C. SPECT scans were acquired with a 30–45 min duration, based on the injected activity, and each SPECT scan was followed by a high-resolution CT scan for coregistration purposes. The SPECT data were reconstructed through an MLEM algorithm, with 250 *μ*m voxel size and 500 *μ*m iterations. Images were decay corrected and normalized between administration routes. CT data were reconstructed through an ISRA algorithm, with 100 *μ*m voxel size.

### 2.7. Quantification from Nuclear Imaging

Quantification estimation is applied on imaging results, where the accumulation in each organ is measured as a percentage of the initial injected activity [31, 32]. This is performed based on the following steps: (i) a series of known activities in different vials (measured through a dose calibrator) are imaged with both imaging systems; (ii) the count rate is recorded through the imaging systems, for each sample, and its known activity is correlated through an activity vs. count rate calibration curve; (iii) based on these curves, the count rate recorded in each organ is translated to activity in each region of interest (ROI) or organ; (iv) this activity/(ID) is divided by the injected dose (i.e., activity), thus providing the %ID/ROI or organ.

For the fast dynamic imaging performed with *γ*-eye™, postprocessing and quantification are performed through the embedded analysis software, visual|eyes™ (BIOEMTECH, Greece). ROIs are drawn on major organs of interest, and then, these ROIs are applied to the individual frames, to provide semiquantitative time activity curves. The count rate per ROI is immediately shown on the post processing of the embedded software, and after a simple division with the injected activity, the %ID/ROI (or organ) is easily and quickly extracted.

For the tomographic images acquired through x-CUBE/y-CUBE™, postprocessing and quantification were performed through third-part analysis software, VivoQuant v1.23 (Invicro LLC, Boston). Volumes of Interests (VOIs) are drawn on major organs of interest, and then, the count rate in a VOI is translated into activity per organ and then divided with the injected activity, to give %ID/organ [33].

## 3. Results

### 3.1. FeHA Characterisation Results

The detailed characterization of FeHA NPs is reported in previously published work by the authors [24]. Briefly, FeHA features a superparamagnetic-like behavior, with a very high net magnetic moment per iron atom (130 emu g^−1^ of Fe), no residual magnetization at room temperature, and a mass magnetization at saturation of 8.7 emu g^−1^ ([34].). FeHA is made of particles having a needle-like morphology with length ranging from 70 to 100 nm and width ranging from 15 to 25 nm, which in turn are composed of smaller aggregated particles of about 5–10 nm in width and 10–20 nm in length. Externally to the needle like particles, electron-dense and round shaped nanoparticles having radius in the 5-10 nm range can be detected by TEM. These NPs were identified as maghemite by Mossbauer spectroscopy, extended X-ray absorption fine structure (EXAFS) spectroscopy, and electron diffraction ([34].).

### 3.2. Radiolabeling Results

**Figure 1:**
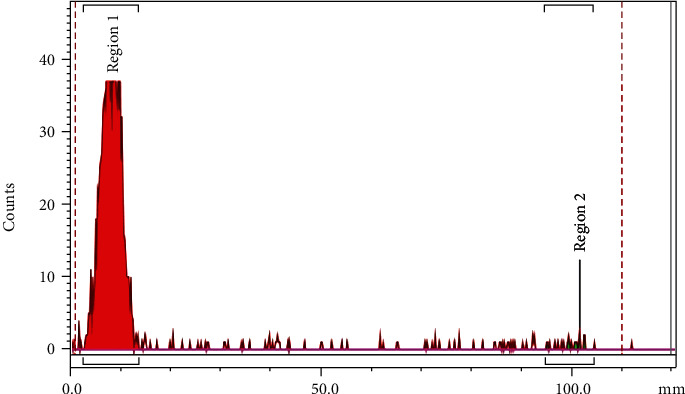
Radio TLC of [^99m^Tc]TcMDP-^Fe^CaPs on ITLC-SG chromatography paper with saline (0.9% *v*/*v*) as eluent.

The labeling of the NPs with ^99m^Tc was assisted by the chelating agent with bisphosphonate arms ([^99m^Tc]TcMDP) that proved to be effective for SPECT imaging. The radiochemical yield for [^99m^Tc]TcMDP-^Fe^CaPs was >95% providing a single radioactive species, with no need of further purification via centrifugation, as detected by ITLC-SG reported in Figure 1.

### 3.3. Proof of Multimodal Functionality for FeHA NPs

The combined results of the MRI and SPECT/CT studies on the same two indicative mice treated by intravenous administration of FeHA NPs are presented here. The first one was imaged with MRI at 10 min p.i. and the second one at 60 min p.i. The protocols used are presented above, and image contrast is quantified through T2 relaxation times, by comparing the values pre- and post-NP administration. SPECT/CT was followed right after the MRI scans, with duration of 1 h and 1.5 h, respectively.

Tables 1 and 2 summarize the T2 relaxation times extracted from the MSME images.

These results show a contrast enhancement of around 41% for 10 min p.i and of 55% for 60 min p.i, when the liver is under study. Relevant values for the FDA approved Endorem® are of 18% p.i.

**Figure 2:**
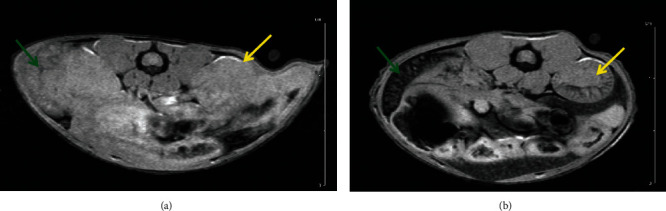
FLASH-kidney-spleen prescan (a); FLASH-kidney-spleen 10 min p.i. (b). Annotation shows the liver (yellow arrows) and the spleen (green arrows).

**Figure 3:**
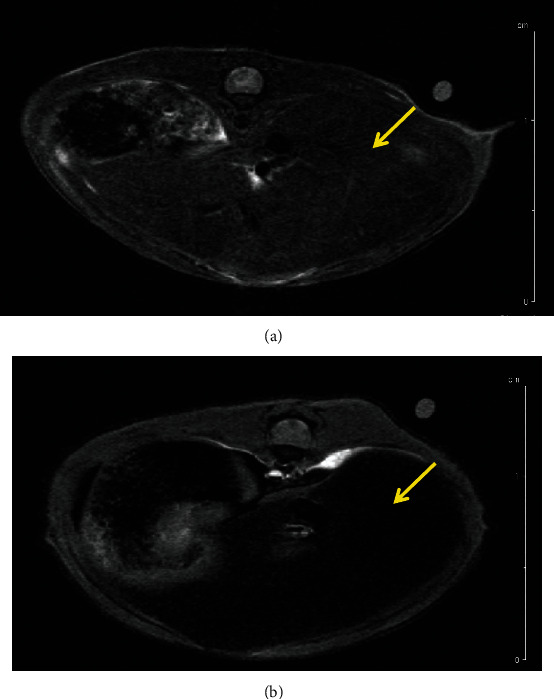
RARE-liver prescan (a); RARE-liver 10 min p.i. (b).

**Figure 4:**
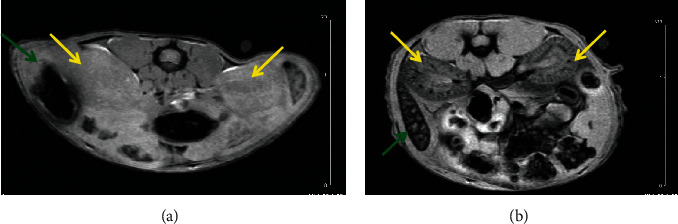
FLASH-kidney prescan (a); FLASH-kidney 60 min p.i. (b). Annotation shows the liver (yellow arrows) and the spleen (green arrows).

**Figure 5:**
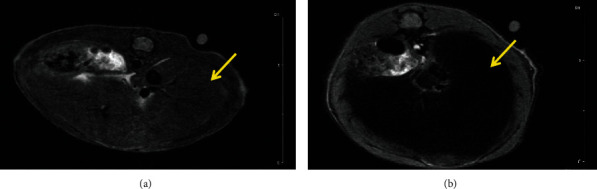
RARE-liver prescan (a); RARE-liver 60 min p.i. (b).

**Figure 6:**
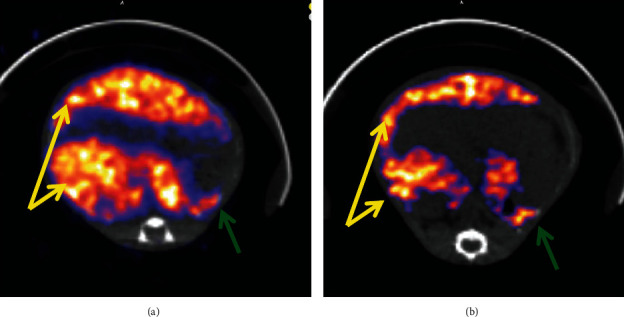
SPECT/CT imaging (NanoSPECT/CT, Mediso) of a mouse at 10 min p.i. (a) and a mouse at 60 min p.i. (b), showing accumulation in the liver (yellow arrows) and in the spleen (green arrows). Both mice were injected iv (tail vein), euthanized at the specific time points, and imaged firstly on SPECT/CT and directly after on MRI (Figures 2–5).

It can be seen that radiolabeled FeHA NPs present an efficient contrast agent for both MRI (Figures 2–5) and SPECT modalities (Figure 6).

### 3.4. In Vivo Molecular Screening

Real-time, *live* dynamic scintigraphic imaging was performed right after administration for each administration route. Successive frames of 2 min duration were acquired for one-hour postinjection, following the kinetics of the NPs. Scintigraphic images are automatically fused with a digital photograph of the mouse, to provide anatomical coregistration.

**Figure 7:**
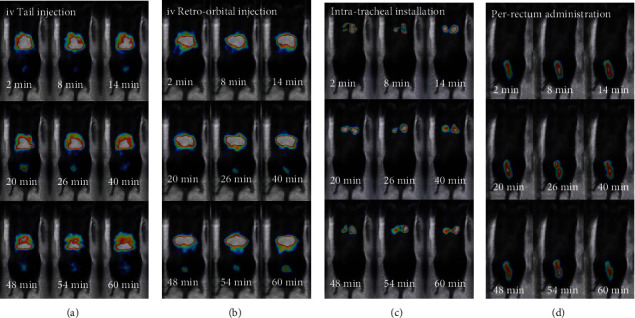
*In vivo* molecular screening for all tested routes with *γ*-eye™, over the first hour p.i.: (a) standard IV tail vein injection, (b) retro-orbital IV injection, (c) intratracheal installation, and (d) per rectum administration. Time point postinjection is shown on each image. All images are decay-corrected. Indicative images are shown (*n* = 5 for each administration route).

**Figure 8:**
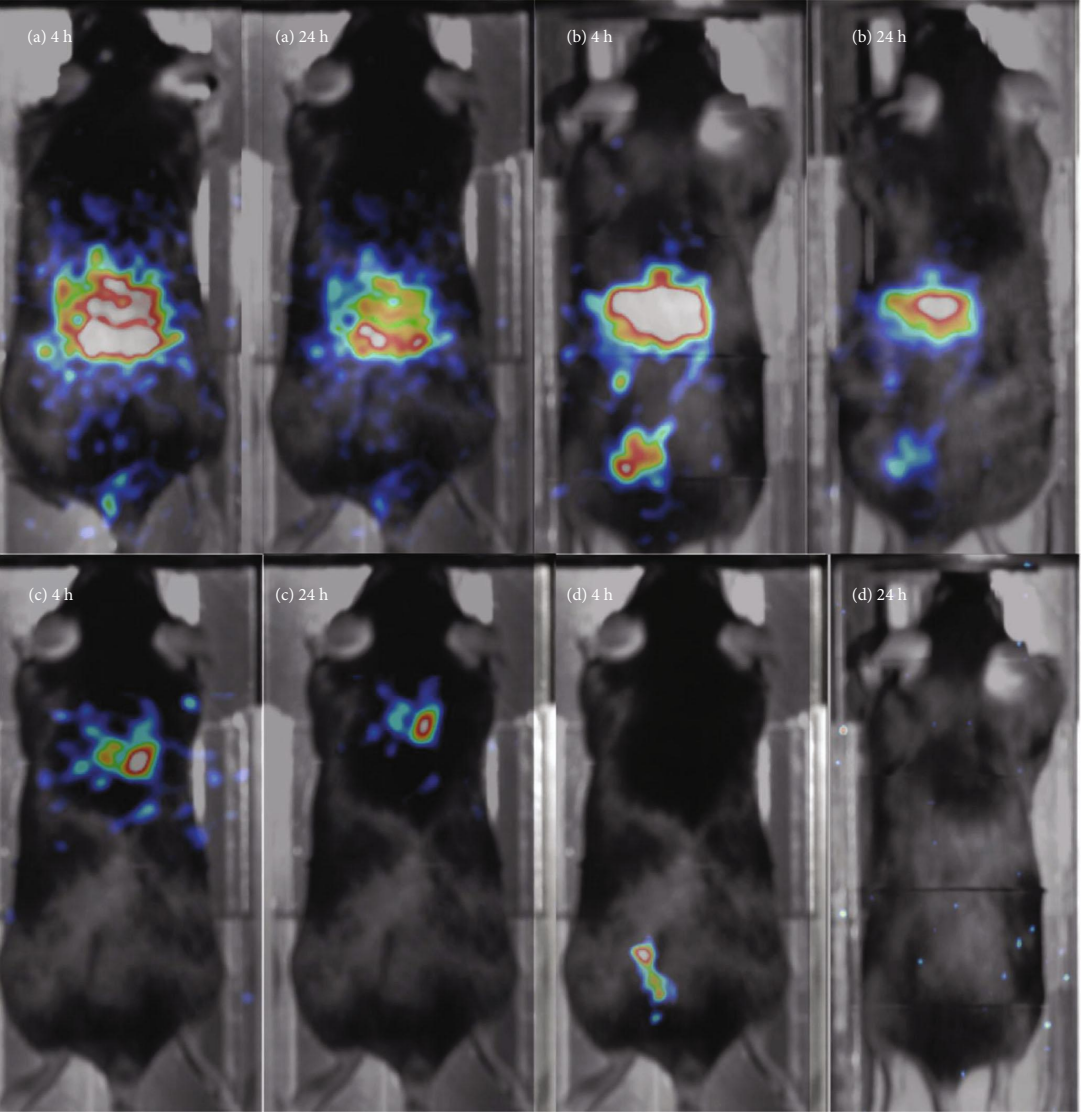
*In vivo* molecular screening for all tested routes with *γ*-eye™, for the 4 hrs and 24 hrs p.i.: (a) standard IV tail vein injection, (b) retro-orbital IV injection, (c) intratracheal installation, and (d) per rectum administration. Time point postinjection is shown on each image. All images are decay-corrected. Indicative images are shown (*n* = 5 for each administration route).

Separate and progressive indicative frames for all the routes are presented in Figure 7 and 8.

These results suggest that these NPs present a very stable body distribution by remaining in the administration region of the body for 24 hours (with an exception of P.R. administration).

Based on these results and the effective targeting effect that can be achieved, tomographic imaging was performed for 1 h, 4 h, and 24 h p.i., for all routes.

### 3.5. SPECT/CT Imaging

**Figure 9:**
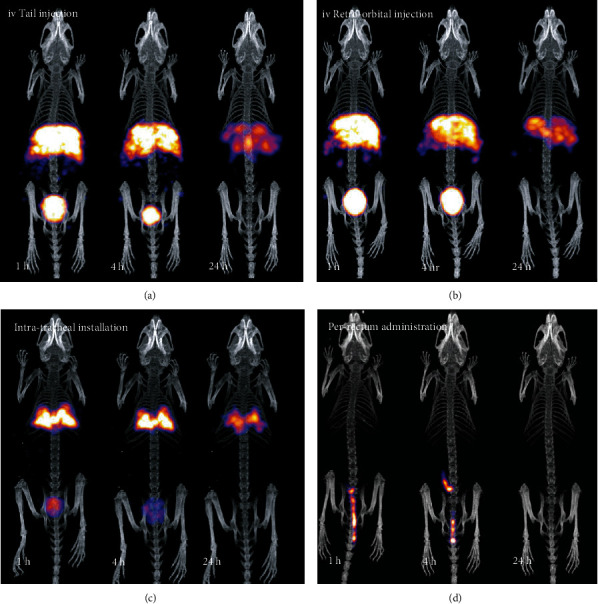
Indicative images of all the administration routes (*n* = 5), for the selected times postadministration, taken with the Molecubes systems: (a) standard IV tail injection, (b) retro-orbital IV injection, (c) intratracheal installation, and (d) per rectum administration. Time point postinjection is shown on each image. All images are decay-corrected.

After the first hour post administration, a tomographic study was initiated, for the mice that were studied through 2D screening (Figure 7). Tomographic fused images are presented on Figure 9. The tomographic study was repeated on each animal at 4 h and 24 h postadministration for all routes under study.

The longitudinal imaging results (Figures 8 and 9) suggest that even after 24 h, the NPs remain in the targeted tissues (i.e., liver, lungs, and colon, respectively). The decay-corrected data show no tissue washout for the period under study. An exception applies to the intrarectal route, where clearance is observed in the first day but still seems to remain in the colon area for more than 4 h. A better uptake could be obtained with animal fasting that would minimize excretions.

This property renders these NPs good targeting molecules, as they present a very high accumulation in the target organ but also have a good and safe absorption due to their biocompatible composition.

### 3.6. Quantification from Images

Quantification from the dynamic imaging studies was performed on visual|eyes™, the software provided along with the eyes™ systems (BIOEMTECH, Greece), as described before.

Uptake in target organs was extracted through visual|eyes™ and analyzed as described above. The %ID/organ is presented in Table 3, for all time points under study.

Quantification from the CUBES™ systems was performed through VivoQuant™ as described in 1.7. The %ID/organ is presented in Table 4, for the selected time points.

These results are also in agreement with previously published *ex vivo* biodistribution data, regarding the tail vein iv injection at the reference time points, published by the authors (Adamiano A 2018).

### 3.7. Overview of Optimized Imaging Protocol

Finally, to investigate the targeting and the therapeutic effects of new promising nanoformulations, we suggest following a stepwise approach:

1. Quick 2D screening with scintigraphic imaging, of 2-3 selected administration routes, based on the desired target area. This allows to identify the most suitable administration route to use in the study
2. Quick 2D screening with scintigraphic imaging, on the preparation conditions (fasting–not fasting), if considered relevant to targeting efficacy. This allows for the optimization of the animal procedure to achieve an optimal targeting effect
3. 2D screening with scintigraphic imaging to find the optimal time points at which the nanoformulation has the best accumulation, according to the desired effect
4. For the selected time point(s), proceed to either *ex vivo* biodistributions or tomographic imaging (if available). This allows to reduce the number of experimental animals and to save time, since only a fraction of the animals will be investigated thoroughly
5. Screening for the animals that are to be either studied through biodistribution or tomographic imaging, to ensure that no bad injection, aggregates or any other undesired effect has occurred. The identified animals, being inappropriate for the study, would be excluded from the study. This will save time and resources

## 4. Discussion

The tested iron-based nanoformulation was already established as a hybrid bimodal MRI/SPECT probe [24] likely to load different biomolecules and was tested to investigate its potential for nuclear planar and tomographic imaging of several target tissues following its delivery via different administration routes. The gold standard to define all biodistribution and targeting parameters in an experiment is still *ex vivo* bio distributions, a method through which mice are sacrificed over a time course and representative tissue samples are collected and analyzed to determine the uptake, usually through gamma-counter measurements [29]. In our proof-of-concept study, the chosen administration route has been proved to significantly affect the biodistribution of the studied NPs. Depending on the desired target region, different amounts of the substance might end up in the target tissue and affect the potential therapeutic result. This effect could be imaged through simple 2D imaging, and 3D scans did not provide additional information. However, 2D scans allowed to obtain a fast first impression of NP kinetics and decide on the next steps for both *ex vivo* biodistributions and 3D imaging. These results and the limits of the methodologies could also be addressed in a disease model, where further parameters could be established and studied.

In the past, multiple studies have been carried out, showing that both planar and tomographic in vivo imaging provide high correlation to *ex vivo* studies and can thus be trusted as an alternative for biokinetics studies [35, 36]. The percentage of an injected substance that reaches each organ can be evaluated with both methodologies, even though there are few parameters like the heterogeneity that are better addressed through tomographic imaging. A detailed study showed that radiopharmaceutical uptake assessed in excised tumours, correlated with that derived from *in vivo* planar (*r* = 0.94, *P* < 0.05, *n* = 18) and SPECT (*r* = 0.90, *P* < 0.05, *n* = 18) images. The biodistribution parameter of percentage of injected dose per gram of excised tumours correlated with the same measure derived from planar (*r* = 0.90, *P* < 0.05, *n* = 18) or SPECT (*r* = 0.87, *P* < 0.05, *n* = 18) images [37]. These results are also reinforced by the results of the present study, regarding time points (1, 4, and 24 hrs) that were studied both on planar dynamic imaging and tomography (Tables 3 and 4). Based on our study, the differences in calculated uptake values between 2D and 3D imaging are around 4.9 ± 3.2%. The suggested imaging protocol and workflow takes advantage of these results to exploit real-time, dynamic scanning for the best choice on multiple parameters (as administration routes, preparation conditions (fasting and heating, etc.), administered dose and activity, and of time points with optimal uptake) before proceeding to *ex vivo* biodistributions and tomographic imaging (i.e., studies for more detailed results), only for the best conditions and selected time points and animals.

The increased interest in the effect of administration routes, specifically for calcium phosphate NPs, is reinforced by relevant recent publications on PET/CT imaging, monitoring NPs for up to 4 hrs postadministration [38]. Due to their superparamagnetic features and its favorable biocompatibility, FeHA has been recently proposed as an alternative to SPIONs for both imaging [24] and hyperthermia applications [39]. In this respect, its diagnostic abilities as T2 negative contrast agent for MRI and as imaging probe for SPECT have been already reported, demonstrating the potential of FeHA for the development of multimodal SPECT/PET-MRI imaging probes. To that respect, additional *in vivo* studies have been performed and presented in this work, to further establish the MRI contrast induction on *in vivo* models and the ability for the exact same dosages and injections to also efficiently work on SPECT imaging. The MRI results showed a contrast induction of 55%, much higher compared to standard MRI contrast agents [24], on a dose that is also suitable for SPECT imaging. Moreover, FeHA has been already used for different nanomedicine applications, such as magnetic labeling of stem cells and for the drug delivery of several anticancer molecules (e.g., doxorubicin and methotrexate) ([34].) [40]. In virtue of its proved diagnostic and drug delivery abilities, we selected FeHA as a nanometric tool to investigate the correlation between different administration routes and NP biodistribution.

The specific FeHA NPs have been studied as a drug-carrier to target specific regions, due to their stability and biocompatibility and the proven fact that they remain in specific regions for period of time (the so called “therapeutic window”) that enables the drug to sort its therapeutic effect. This characteristic could also be exploited by slow infusion and gradual release of pharmaceutical in the region of interest [41], which is possible with the suggested workflow and the use of real-time 2D imaging. Our results suggest that these NPs present a very stable behavior by remaining in the administration region of the body for the first hour, rendering them an appropriate candidate for drug targeting in multiple body tissues.

The administration routes that have been studied provide a wide range of possible popular target tissues, for plenty of diseases. The iv route through the tail vein is the most popular administration route used for *in vivo* studies on mice. Many papers have already demonstrated that through this administration route, a high amount of nanoparticles can reach the liver and boost hepatic therapy schemes for fibrosis or cancer [42–44] and also the need of the probe to be eliminated from healthy tissues in a relevant short period. The same concept is applicable for iv administrations through the retro-orbital vein that was found to provide the same biodistribution to FeHA NPs, with the added advantage of minimum stress for the animals [28]. On the other hand, fast absorbance by the liver may be a limiting factor in other applications where higher blood circulation times are required.

Regarding the it installation, there are many lung diseases that could be targeted with this method, such as pulmonary fibrosis or lung cancer [45, 46]. For this type of diseases, iron-free calcium phosphate NPs have already been studied to abate pulmonary inflammation [47]. Moreover, it administration of calcium phosphate NPs was recently proved to be an effective strategy to increase the efficacy of inhalation-bases therapy for the treatment of heart diseases [48]. Lastly, the rp administration can target colon and colorectal for the treatment of diseases like cancer, inflammation, or other disorders [49–51] that are typically very difficult to target using other administration routes. The uptake of this method could be further optimized by firstly clearing the colon, as regardless of whether the mice have fasted; there is always the possibility of the presence of feces in the colon that limits the space for administration of a given volume [52].

Since by following the proposed workflow, it is possible to test more parameters with less time and in fewer animals (administration route, administered activity and dosage, fasting conditions that improve uptake, etc.); its application will allow the optimization of future studies in terms of number of animals, time, and economical resources. Furthermore, our method allows for a fast control of the experimental set up, since any unsuccessful administration or sample malfunction (i.e., formulation of aggregates) can be immediately spotted and the specific animal can be excluded from the study with no further study time wasted.

In this way, a study is performed on more robust parameters that can possibly reduce the number of experimental cycles and thus the requested animals, research time, and associated costs. The best time points (best targeting effect) can also be easily decided on 2D imaging, and tomographic imaging or *ex vivo* biodistributions (i.e., studies for more detailed results) can be performed only for these time points, reducing the total number of animals in the study and thus in compliance with the 3Rs principle in animal research [53]. Finally, the proposed set up can be further explored as a fast readout when a characterization of modified NPs (e.g., surface functionalization with targeting moieties) in comparison to the pristine NPs is required.

## 5. Conclusion

A molecular imaging protocol able to optimize *in vivo* studies, reduce significantly experimental time, animal needs, and research cost, able to provide more sound and rigorous results, has been presented. By performing initial fast tests on *live*, real-time dynamic screening, multiple parameters are defined, and then, more detailed studies through *ex vivo* biodistributions and tomographic imaging can be performed based on more robust parameters and on selected time points and animals. This concept was demonstrated using a new biocompatible iron doped hydroxyapatite NP formulation that is also introduced and further studied, to act as carrier to specifically target multiple region of interest, due to its high stability and high targeting effect.

## Figures and Tables

**Table 1:** Prescan and 10 min p.i.

	Pre (msec) ± SD	10 min p.i.(msec) ± SD
Liver	18.5 ± 0.9	10.8 ± 0.5
Spleen	27.5 ± 0.8	18.3 ± 0.7
Kidneys	35.1 ± 2.2	29.4 ± 1.7

**Table 2:** Prescan and 60 min p.i.

	Pre (msec) ± SD	60 min p.i.(msec) ± SD
Liver	20.9 ± 3.9	9.4 ± 0.4
Spleen	26.4 ± 0.9	17.7 ± 0.5
Kidneys	30.1 ± 2.7	21.3 ± 0.8

**Table 3:** % ID per organ calculated through visual|eyes™, for the target organs of each administration route, based on the images presented on Figure 7. Average values ± SDs are presented, based on minimum 2 animals per time point.

Administration route:		iv	ro	it	pr
Target organ:		Liver	Liver	Lungs	Colon
%ID/organ ± SD	2 min	49.3 ± 6.6	55.5 ± 4.7	86.4 ± 3.1	85.2 ± 4.5
	20 min	52.9 ± 3.4	59.7 ± 6.8	87.3 ± 8.2	86.8 ± 2.8
	40 min	56.9 ± 4.2	66.6 ± 7.3	87.5 ± 7.1	85.7 ± 3.4
	60 min	57.1 ± 5.2	58.7 ± 8.6	80.8 ± 5.8	81.0 ± 5.8
	4 h	62.0 ± 4.3	67.5 ± 5.6	57.1 ± 5.2	39.8 ± 4.6
	24 h	37.5 ± 7.4	33.8 ± 6.4	42.9 ± 4.6	0.0 ± 0.5

**Table 4:** % ID per organ calculated through VivoQuant™, for the target organs of each administration route, based on the images presented on Figure 9. Average values ± SDs are presented based on minimum 2 animals per time point.

Administration route:		iv	ro	it	pr
Target organ:		Liver	Liver	Lungs	Colon
%ID/organ ± SD	1 h	56.1 ± 7.1	63.6 ± 12.9	73.5 ± 8.3	79.7 ± 12.4
	4 h	63.4 ± 6.6	66.6 ± 7.5	63.4 ± 6.7	35.9 ± 9.2
	24 h	39.7 ± 6.5	31.8 ± 5.2	43.8 ± 4.9	0.5 ± 0.8

## Data Availability

The underlying data supporting the results of our study can be found in a private server that can be accessed through dedicated passwords.
